# Localization of a contraceptive implant using a silicon chip-based handheld ultrasound device

**DOI:** 10.1007/s00404-025-08045-z

**Published:** 2025-06-24

**Authors:** Ruben Plöger, Eckhard Binder, Brigitte Strizek, Adeline Walter, Florian Recker

**Affiliations:** 1 Department of Obstetrics and Prenatal Medicine, University Hospital Bonn, Venusberg Campus 1, 53127 Bonn, Germany; 2 Department Eidinghausen, GYNCOLLEGWESERLAND, Eidingsen 2, 32549 Bad Oeynhausen, Germany

**Keywords:** POCUS, Ultrasound imaging, Contraception, Gynecology

## Abstract

**Background:**

Etonogestrel contraceptive implants (Implanon/Nexplanon) are a highly effective form of long-acting reversible contraception. Removal is typically straightforward when the implant is palpable in the upper arm. However, it can become challenging if the implant cannot be located by palpation due to deep placement, weight gain, or migration. Ultrasound is the recommended first-line imaging modality to localize non-palpable implants. Standard cart-based ultrasound devices may not be readily available in all settings (e.g., outpatient clinics, operating rooms, or resource-limited regions). A new generation of handheld ultrasound devices based on silicon chip technology has recently emerged, offering high-resolution imaging in a handheld, point-of-care format. These devices have the potential to facilitate rapid bedside localization of implants.

**Methods and results:**

We present the first case of localizing a non-palpable Implanon NXT® (68 mg etonogestrel) rod using a silicon chip-based handheld ultrasound device (Butterfly iQ). A 27-year-old woman with a non-palpable upper arm implant underwent sequential scanning with the handheld ultrasound device connected to a smartphone and with a standard piezoelectric ultrasound device for comparison. The handheld ultrasound readily identified the implant as an echogenic focus in the arm, enabling marking of the location for removal. A confirmatory scan with a standard ultrasound unit likewise visualized the implant. Guided by these imaging findings, a small incision was made directly over the implant site and the rod was removed successfully under local anesthesia.

**Conclusion:**

This case demonstrates that a semiconductor-based handheld ultrasound can reliably detect a non-palpable contraceptive implant, yielding sonographic images comparable to a standard piezoelectic ultrasound device. The successful localization and removal of the implant using the portable device suggests that this new ultrasound technology can be a valuable tool in obstetric and gynecologic practice for managing challenging implant cases. Its portability and ease of use at the point of care may improve access to timely implant removal in clinic settings, operating theaters, and remote or underserved areas. Wider adoption of this technology, alongside formal studies validating its accuracy, could enhance clinical workflows for contraceptive implant management.

## What does this study add to the clinical work

**Table:** 

Handheld ultrasound based on silicon-chips can be used to detect a non-palpable implanon in situations without access to a standard ultrasound device. When used, the handheld ultrasound device demonstrates its advantages such as being location-independent and flexible in time.	

## Introduction

Contraceptive implants are a widely used form of long-acting reversible contraception, valued for their high efficacy and convenience [1]. The etonogestrel implant (Implanon NXT®/Nexplanon®) consists of a single 4 cm by 2 mm semi-rigid rod and is placed subdermally in the inner upper arm. It provides at least 3 years of continuous contraception and is considered highly effective and safe [1]. Reported pregnancy rates are exceedingly low, and its effectiveness is comparable across different body weights [2]. Common indications for early removal (before the 3-year duration) include troublesome bleeding patterns (the most frequent side effect), desire for pregnancy, or no longer needing contraception [3]. In routine practice, if the implant is easily palpated, removal can be performed with a minor outpatient procedure through a small incision under local anesthesia. Challenges arise when an implant is non-palpable. Non-palpability can occur due to an insertion that is deeper than intended, weight gain with an increase of subcutaneous fatty tissue or due to migration of the implant from its original position. An estimated 0.1% (1 in 1000) of insertions result in an implant placed too deep to feel on exam. “Migration” of an implant is defined as movement of more than about 2 cm from the initial insertion site [3–5]. Minor shifts are not uncommon, with the axillary region being the most frequent site if the implant moves within the arm. In rare cases, serious complications have been documented, such as intravascular migration of the implant into the pulmonary arterial circulation or impingement on neurovascular structures. A recent systematic review identified 148 cases of ectopic implant migrations in the literature (74 into pulmonary vessels and others into distant sites) [4], highlighting that, while rare, these events can occur and pose significant health risks. Such cases underscore the importance of accurately locating a non-palpable implant before attempting removal. Ultrasonography is the first-line imaging modality for localizing non-palpable contraceptive implants [3, 6]. High-frequency linear array probes (typically 10–15 MHz or higher) are recommended to visualize the small rod under the skin [3]. The implant itself is echogenic relative to soft tissue, appearing as a bright linear echo when the transducer is aligned along the rod’s long axis, or as a punctate bright dot on transverse scans. A posterior acoustic shadow is often seen behind the implant due to the density difference (especially with Implanon/Nexplanon, which contains barium to increase radiopacity) [3, 5, 6]. Confirmation of an implant on ultrasound is strengthened by visualizing it in two orthogonal planes and noting the characteristic shadowing, which helps distinguish the implant from linear echoes of fibrous septa in the arm [6]. Once the implant is localized by ultrasound, removal can be performed immediately under ultrasound guidance or after marking the skin over the implant. Ultrasound-guided removal is highly successful: one specialized center reported that ultrasound could localize 98% of non-palpable implants, enabling office removal of the majority of them [7]. Moreover, even deeply situated implants that require surgical removal can be removed with minimally invasive techniques under continuous ultrasound visualization, with high success rates and safety [8]. In standard practice, if an implant is not located with ultrasound, additional imaging modalities are employed. For implants that contain radiopaque markers (like Nexplanon/Implanon NXT), a plain X-ray of the arm can often reveal the implant’s location [6]. If the implant remains elusive (for example, non-radiopaque implants like older Implanon or Jadelle, or if an X-ray is inconclusive), magnetic resonance imaging (MRI) of the arm and chest can be utilized, especially to detect deep or intravascular migrations [6]. Ultimately, if imaging suggests migration to the pulmonary artery, a chest CT scan will confirm it, and an interventional radiology or surgical procedure is required for removal [6, 9]. An algorithm outlining the recommended approach to localize non-palpable implants is presented in Fig. 1.

**Figure:**
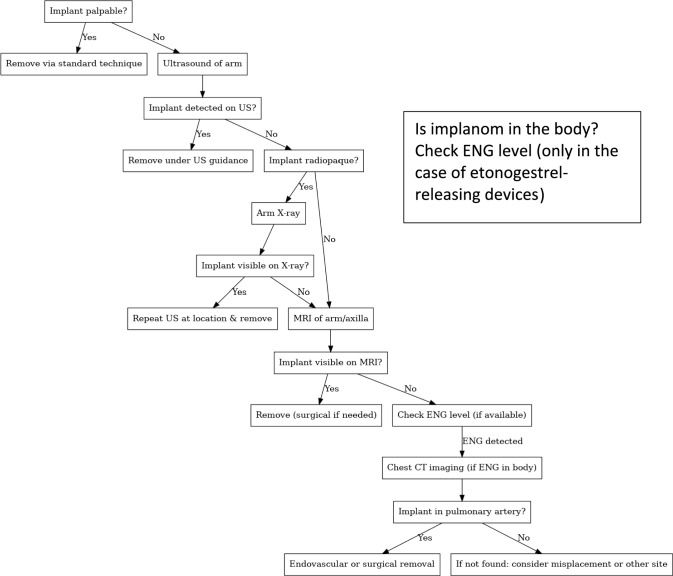
Decision-tree for localization and management of a non-palpable contraceptive implant. CT: computer tomography ENG: etonogestrel, MRI: magnetic resonance imaging, US: ultrasound examinataion

While ultrasound is highly effective for implant localization, one practical limitation is the availability of ultrasound equipment at the exact time and place of patient care. Standard ultrasound devices are typically cart-based or console units that may not be immediately accessible in a clinic exam room or minor procedure room. Recently, however, handheld ultrasound devices have revolutionized point-of-care imaging. These handheld units use semiconductor transducers (such as capacitive micromachined ultrasound transducers, CMUTs) in place of traditional piezoelectric crystals [10]. The Butterfly iQ device, for example, contains an array of 9,000 micro-machined sensors on a silicon chip, covering a broad frequency range and plugged into a smartphone for image display [10]. This “ultrasound-on-a-chip” approach dramatically reduces the size and cost of the devices while maintaining imaging capabilities comparable to standard ultrasound devices. Unlike standard ultrasound devices that often require swapping probes for different applications (linear, curvilinear, cardiac, etc.), the silicon chip probe of the handheld ultrasound devices can perform multiple functions by electronically adjusting its output frequency and beam parameters. These handheld ultrasound devices have been successfully introduced into many medical fields, including emergency medicine, critical care, and gynecology and obstetrics [11–16]. They enable clinicians to carry an ultrasound in their coat pocket or bag and perform examinations in almost any setting—from bedside assessments in the office to triage in low-resource environments [10]. In obstetrics and gynecology, studies have demonstrated that modern handheld ultrasound devices can be used to accurately perform tasks such as fetal biometry and gestational age estimation, comparable with highend standard ultrasound devices [13, 15, 16]. In studies, the measurements of fetal size obtained by a handheld ultrasound device (Butterfly iQ) showed excellent agreement when compared with standard ultrasound devices (intraclass correlation ≥ 0.95), confirming these devices’ suitability for clinical use [13, 15, 16]. Similarly, evaluations of handheld ultrasound devices in postpartum care and gynecologic scanning have shown high reliability and diagnostic accuracy [12, 14].

Given this technological advancement, we hypothesized that a handheld silicon chip-based ultrasound device could be used to localize a non-palpable contraceptive implant at the point of care. This report describes the first such application and compares the performance of a handheld ultrasound device to a standard ultrasound device in the context of implant removal.

## Methods

The patient was a healthy adult woman who provided informed consent for the examination and for publication of her case details. The study was performed in accordance with the Declaration of Helsinki and was approved by the local institutional review process.

For ultrasound examinations, we used a silicon chip-based handheld ultrasound device (Butterfly iQ+, Butterfly Network, USA) and a standard cart-based ultrasound device (Samsung HS40, South Korea) equipped with a high-frequency linear transducer. The Butterfly iQ+ is a handheld probe that connects to a smartphone and contains a two-dimensional matrix of CMUT elements, allowing both high-frequency (for superficial imaging) and lower frequency (for deeper structures) ultrasound imaging through the same probe [10]. The HS40 is a conventional ultrasound system utilizing piezoelectric transducer technology and was used with a 5–13 MHz linear array probe for soft tissue imaging. Before the exam, the handheld ultrasound device was calibrated via its mobile application and set to a “small parts” preset (to optimize visualization of superficial structures). The standard ultrasound device was set to an equivalent preset for soft tissue. Both devices were used with generous application of acoustic gel on the patient’s arm. The patient was placed in a supine position with her right arm (non-dominant arm, containing the implant) abducted about 90°—a position exposing the inner aspect of the upper arm as typically recommended for implant insertion and localization. First, the scan using the handheld ultrasound device was performed. The probe was moved slowly along the area of the insertion scar and surrounding tissue in both longitudinal and transverse orientations, while the operator observed the real-time image on the smartphone screen. Once an object consistent with the implant was identified, its location and depth were assessed by adjusting the probe angle and using on-screen calipers. We defined a positive identification as visualization of a linear or punctate echogenic structure with dimensions ~4 cm by 2 mm, distinguishable from connective tissue echoes, and present in both transverse and longitudinal views. After the ultrasound examination using the handheld ultrasound device, a second ultrasound examination was performed using the standard ultrasound device for comparison. The same steps were repeated with the linear array probe of the HS40, by a different operator who was blinded to the exact location marked from the hand-held scan. Still images from both the portable and standard ultrasound were saved, and the visibility of the implant (and any acoustic shadowing) was noted for each device.

Following imaging, the skin overlying the implant was marked (based on the ultrasound findings) to guide the removal procedure. The removal was then carried out by the gynecologic surgeon in the outpatient minor procedure room. The skin was cleaned and infiltrated with 1% lidocaine anesthetic at the marked site. A small 4 mm incision was made directly over the distal end of the implant (as identified on ultrasound), and the implant was carefully dissected and extracted using standard techniques (gentle blunt dissection and traction with a hemostat).

## Case presentation

### Patient information

The patient was a 27-year-old G0P0 (gravida 0, para 0) woman who presented for removal of her etonogestrel contraceptive implant. The implant (Implanon NXT®) had been placed in her left upper arm approximately 3 years prior. She sought removal at the end of its 3-year effective lifespan to potentially plan for pregnancy. She reported no major issues while the implant was in place aside from mild irregular bleeding, and her medical history was otherwise unremarkable. Importantly, she stated that she could no longer feel the implant under her skin, which prompted concern about its position. There was no history of implant expulsion or previous attempt at removal.

### Clinical examination

On examination of the inner aspect of the left upper arm, a small insertion scar was noted about 8 cm proximal to the medial epicondyle of the humerus (just above the elbow). On palpation, the implant rod was not detectable. There was no tenderness or edema in the area. Given the non-palpability of the device, an imaging localization was indicated prior to removal.

### Ultrasound examination

A ultrasound scan was performed bedside using the Butterfly iQ+ device. The implant was successfully visualized on the handheld ultrasound device. On longitudinal scanning along the axis of the arm, the implant appeared as a thin echogenic line within the subcutaneous tissue of the arm, approximately 3 mm beneath the skin surface. On transverse (short-axis) view, it appeared as a bright dot about 2 mm in diameter. The ultrasound images showed the rod and allowed estimation of its depth and orientation. Notably, the implant did not produce a strong acoustic shadow—the tissue behind the bright rod appeared only mildly attenuated. Despite the minimal shadowing, the implant’s echogenicity and shape were distinctive enough to confirm its identity. We then performed a confirmatory scan with the standard piezoelectric ultrasound device. Using the Samsung HS40 with a linear probe, the implant was again clearly identified in both longitudinal and transverse planes. The examination using the standard ultrasound device showed an obvious linear echo corresponding to the rod, and in this modality a posterior acoustic shadow was apparent behind the implant. Aside from the difference in shadowing, the two devices produced comparable imaging of the implant. Both ultrasounds indicated that the implant was located in the subcutaneous fat layer above the fascia and slightly more deeper than the usual placement. No surrounding hematoma, fibrosis, or abnormality was seen. The tip of the implant was approximately 7 cm above the palpable bony landmark of the medial epicondyle, slightly proximal to the insertion scar (suggesting minimal cephalad migration of a few millimeters). These findings gave the surgical team confidence in proceeding with removal at the precisely identified location.

### Implant removal

With the implant’s position marked on the skin, removal was performed under local anesthesia. A 4 mm transverse skin incision was made directly over the distal end of the implant as guided by the ultrasound markings. Using blunt dissection with a small hemostat through the incision, the implant was located and grasped. The distal tip of the rod was gently advanced out through the incision (“pop-out” technique), and the entire 4 cm implant was extracted intact. The procedure was uncomplicated, with minimal bleeding. A physical inspection confirmed the removed rod was complete and not broken. Steri-strips were applied to close the incision, and a pressure bandage was placed. The patient tolerated the procedure well and reported immediate relief knowing the implant was removed. She had an uneventful recovery with the small incision healing normally at follow-up. The implant was confirmed by lot number as the Implanon NXT placed three years prior.

### Comparative device performance

This case allowed a side-by-side comparison of a handheld ultrasound device and a standard ultrasound device for implant localization. Both modalities successfully detected the non-palpable implant, corroborating each other’s findings. The key difference noted was the presence of a clear acoustic shadow on the conventional ultrasound images, whereas the handheld ultrasound images showed little to no shadow behind the implant. Despite this difference, the primary sign—the brightly echogenic implant itself—was evident with both technologies. The slight migration (a few millimeters) from the scar was easily tracked with ultrasound, whereas it had been impossible to identify by palpation alone.

## Discussion

This case illustrates a practical solution to the problem of a non-palpable contraceptive implant: the use of a next-generation handheld ultrasound device to accurately locate the implant. The successful identification and removal of the implant using the silicon chip-based ultrasound device confirm that this new handheld technology can visualize the characteristic sonographic features of an Implanon rod. In our patient, the implant’s appearance on the portable scanner was consistent with known ultrasound signatures of etonogestrel implants—an echogenic linear structure in longitudinal views and a bright point in transverse views [6]. We were able to appreciate the ends of the rod and its relative depth, which are critical details for planning the incision and removal. A minor difference was observed in that the examination using the handheld ultrasound device did not display a prominent acoustic shadow from the implant, whereas the standard ultrasound device did. Acoustic shadowing is a secondary sign that can help confirm the presence of a dense foreign body [6], but it is considered an *optional* (not always present) finding for implants. Prior reports have noted that not all implants will cast a clear shadow, especially if the ultrasound settings or the implant’s composition reduce the shadow effect [17]. In our case, the absence of a strong shadow on the Butterfly iQ+ images did not hinder identification, since the primary echo from the rod was distinct. It is possible that differences in the device’s gain, frequency, or processing algorithms accounted for the reduced shadow. The portable scanner’s CMUT-based transducer has a broad bandwidth and potentially different artifact profiles than piezoelectric probes, which might explain this variance. Importantly, both ultrasound modalities allowed confident localization of the implant, demonstrating that the handheld device’s image quality was sufficient for this task.

Our experience adds to the growing body of evidence that point-of-care ultrasound can be effectively applied in gynecologic procedures. Portable, handheld ultrasound devices have already been tested in obstetrics and gynecology for applications like fetal growth monitoring, third-trimester scanning, and postpartum evaluation [12–16]. They have shown impressive accuracy, often matching the performance of traditional devices for measurements and diagnostic findings [13, 16]. This case report extends those findings to the niche scenario of contraceptive implant localization. To our knowledge, this is the first reported case of using a semiconductor ultrasound probe to find an impalpable implant in vivo. The implications are encouraging: a gynecologist equipped with a handheld ultrasound can quickly localize an implant during a clinic visit or even in the operating room just before removal, without needing to schedule a separate radiology appointment. This immediacy can reduce delays in care and patient anxiety. It may also eliminate the need for more costly investigations in many cases. For instance, if a portable ultrasound is readily available and confirms the implant’s location, one might avoid having to obtain an X-ray or MRI, which would have been the next steps if the implant remained unlocated [6]. There are several advantages to using portable ultrasound for this purpose. Mobility and accessibility are foremost: the device can be brought to the patient, whether in a clinic exam room, at bedside in a hospital, an operating theater, or a remote field clinic. In the context of implant removals, this means that even if a patient presents to a primary care setting or a small clinic without a full ultrasound unit, a trained provider could still perform the localization on the spot. This capability is especially valuable in low-resource settings or rural areas where standard ultrasound devices are scarce. By facilitating on-site detection, portable ultrasounds can help ensure implants are removed on time (e.g., at the end of their lifespan or when desired) rather than being left in situ due to logistical barriers in locating them. Additionally, the real-time guidance from a handheld ultrasound device can improve the safety of removal procedures. As highlighted in prior studies, deeply placed implants or those near neurovascular structures benefit from ultrasound-guided retrieval to prevent complications [6, 7]. A handheld device can be used intraoperatively, with a sterile sheath over the probe, to directly visualize the implant and surrounding anatomy during dissection. This could reduce the risk of blind exploration, nerve injury, or extensive incisions. In our case, knowing the depth and exact position of the implant allowed us to make a very small incision precisely over one end of the rod, minimizing tissue trauma. The handheld ultrasound device was small enough to be manipulated easily in the tight procedure environment, an advantage over bulkier devices.

This new generation of handheld ultrasound devices provided adequate resolution for visualizing the small implant. This underscores the progress in ultrasound-on-chip technology—earlier generations of handheld ultrasound devices (often using traditional crystals in miniaturized form) sometimes suffered from lower image resolution or depth penetration. The device used here (and similar modern models) have largely closed the gap with standard equipment by using thousands of elements and advanced signal processing [10]. As a result, the image clarity in superficial soft tissue (where implants reside) is high and is used in other specialites as ophthalmology [10]. For implant localization, which requires differentiating a 2 mm object in subcutaneous tissue, our case demonstrates that the resolution was indeed sufficient.

**Fig. 2:**
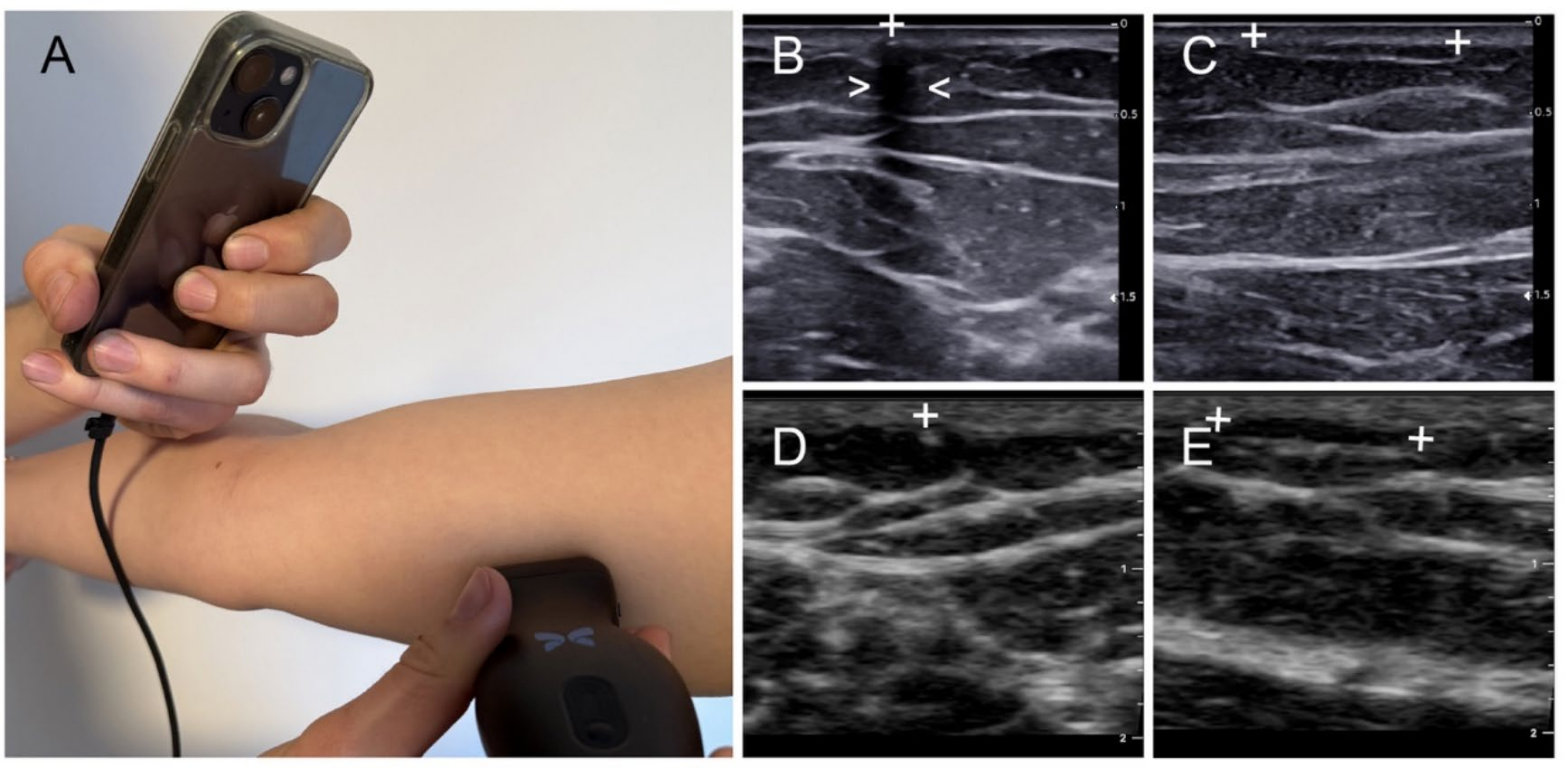
Image of an examination with a handheld ultrasound device (**A**) and of contraceptive implant using a standard ultrasound device based on piezo technology (**B** and **C**) and a handheld ultrasound device based on silicon chips (**D**and **E**): The hyperechoic round region of the contraceptive implant in the transverse scan is marked by the plus and the acoustic shadow is marked by the arrow (**B** and **D**). The hyperechoic line of the contraceptive implant in the longitudinal scan is marked between the plus signs (**C** and **E**)

Despite these advantages, some limitations and considerations deserve mention. First, this report is a single case—broader conclusions require caution. While it shows feasibility, the performance of handheld ultrasound devices for implant detection should be evaluated in a larger series. Factors such as operator experience and patient habitus could influence success. Our operator was experienced in ultrasonography; less trained clinicians might find it challenging to interpret the images, especially given potential artifacts. Misidentification is possible—for example, linear fibrous bands in the arm can mimic the appearance of an implant if one is not careful [6]. Training modules for handheld ultrasound devices [18] or reference images (such as Fig. 2) could help users learn to recognize true implants versus false echoes. Patient body habitus is another factor: in a very obese arm, the implant might lie deeper, pushing the limits of a handheld device’s penetration power. Conversely, in extremely thin patients, implants might lie just under fascia or muscle (subfascial placement) [7], which could also be more challenging to visualize and remove. Another consideration is that the handheld ultrasound device we used did not incorporate Doppler imaging in our examination (though the device is capable of color Doppler). Color Doppler was not necessary for our case since the implant is an inert object, but one could theoretically use Doppler to ensure the bright structure is not a vessel (by checking for absence of blood flow). This may be a useful tip in unclear cases—and portable units today do offer color flow modes to facilitate such checks.

From a technological perspective, the differences between piezoelectic ultrasound devices and silicon-based ultrasound devices might lead to subtle variations in imaging. The lack of acoustic shadowing we saw is one example. It would be worthwhile in future investigations to examine whether the handheld device has any limitations in detecting implants under certain conditions—for instance, very deep implants or implants surrounded by fibrotic tissue might be harder to visualize. Additionally, one must consider cost and availability: while substantially cheaper than a full-sized standard ultrasound device, handheld ultrasound devices still require an investment (typically a few thousand US dollars plus a software subscription). Clinics will need to weigh this cost against the benefits of having immediate ultrasound access. In many cases, the cost is justified given the device’s versatility (it can be used for many clinical purposes beyond just implants). As manufacturing of these semiconductor probes scales up, costs are expected to decrease, making them even more accessible worldwide [10].

This case also highlights an important workflow aspect: using a handheld ultrasound encourages the clinician to perform the scan themselves at point of care, rather than referring the patient to another department. This integration of ultrasound into the clinical exam by the provider (often termed the “physical exam 2.0”) can enhance diagnostic speed. However, it requires that clinicians maintain ultrasound skills and that they operate within their scope of expertise. Obstetrician–gynecologists are generally trained in basic ultrasound, especially in obstetrics, but localizing an arm implant is a relatively straightforward skill to acquire. Our report may encourage gynecologists to consider handheld ultrasound as part of the armamentarium for office procedures. In the future, we envision a protocol where if an implant is not immediately palpable on exam, the clinician can reach for a handheld ultrasound, spend a few minutes localizing the device, and proceed directly to removal—all in one visit. This could prevent scenarios of patients being lost to follow-up when referred elsewhere for imaging, and it would shorten the time the patient has the implant in situ when it is no longer desired or indicated.

The adoption of handheld ultrasound for implant localization could improve patient care by enabling immediate, on-site diagnosis and management (Table 1). Patients with non-palpable implants often experience anxiety and may require multiple appointments (one for imaging, another for removal) [7]. By consolidating these steps, we can provide faster reassurance and definitive treatment. Furthermore, ensuring timely removal of implants that are due to expire is important to prevent unintended prolonged contraception or difficult removals later (implants tend to become more firmly encapsulated by fibrous tissue over time). In cases of deeply embedded implants, having ultrasound at hand allows the practitioner to decide promptly whether an office removal is feasible or whether referral to a specialized center (or surgical removal under anesthesia) is necessary [7]. In the present case, the implant was in a favorable suprafascial plane and was removed in-office. Had the ultrasound shown it to be deeply intramuscular or near major nerves, we might have opted for removal in an operating room with backup from other specialties. Thus, handheld ultrasound devices not only find the implant but also influence the decision-making and planning for a safe removal approach.

**Table 1:** Comparison of standard versus handhled ultrasound devices

Feature	Standard piezoelectric ultrasound	Silicon chip-based handheld ultrasound
Transducer technology	Piezoelectric crystals when electrically stimulataed producing ultrasound Specific frequency ranges in specific probes	Semiconductor CMUT array on a chip (thousands of microscopic membranes) producing ultrasound Broad frequency bandwidth in one probe
Form factor	Console or cart-based unit with separate transducer probes Not easily portable (transport via wheeled cart)	Smartphone/tablet with a hand-held probe (~Butterfly iQ size) Highly portable (pocket-sized)
Image quality	High-resolution imaging for all deepth Gold-standard Advanges options (e.g., harmonic imaging, high-end Doppler modes)	High resolution for superficial and medium depth imaging; Approaching parity with standard devices for many applications Perhaps slightly reduced image quality in very deep fields or specific modes, but continually improving
Imaging modes	B-mode, M-mode, Doppler (color, power, spectral), 3D/4D, etc., depending on model. Extensive processing capabilities on-board.	B-mode and basic Doppler modes (color/power) typically available; some devices offer M-mode. Advanced modes (3D/4D) not usually present. Processing partly offloaded to mobile app/cloud
Use in implant localization	Effective with high-frequency linear probe; clear visualization of implant and shadow if in superficial range	Effective with built-in high-frequency capability; demonstrated ability to visualize implant (this case)
Advantages	Established in clinical practice Familiar interface for sonographers Consistently excellent image quality and reliability	Extreme portability and quick deployment Lowers barrier to performing ultrasound at point of care Serving multiple purpose with one probe Lower cost than standard ultrasound devices
Limitations	Lack of portability Need of space and electric power High cost to purchase Not easily accessible in resource-limited settings	Learning curve with new interfaces Reliance on mobile device battery and software updates Subscription service Slightly limited field of view compared to some specialized probes Minor trade-offs in image fidelity

It summarizes key differences and similarities between a conventional piezoelectric ultrasound unit and the silicon chip-based handheld ultrasound, in the context of implant localization and general use in obstetrics/gynecology [6].

### Potential limitations and future directions

While our case demonstrates feasibility, larger studies should formally evaluate the sensitivity and specificity of handheld ultrasound devices for implant localization. It would be useful to know, for example, if the detection rate approaches similar rates as with standard ultrasound devices [7] and if any implant characteristics (depth, angle, patient factors) reduce detection with the handheld device. A prospective trial could compare time-to-localization using handheld vs standard devices, or compare success rates in the hands of gynecologists vs radiologists. Additionally, further improvements in handheld ultrasound technology are anticipated. Future iterations may incorporate even higher element counts, better resolution, and perhaps automated algorithms. We can envision an algorithm in the ultrasound software that could potentially recognize the hyper-echogenic implant and mark it (using artificial intelligence trained on images), assisting less experienced users. Another future direction is integrating the device usage into clinical protocols—for example, training family planning providers in basic ultrasound use specifically for implant cases, much like how obstetric providers learn ultrasound for pregnancy. Given that implants are often placed and removed by trained nurses or mid-level providers in some settings, having an accessible ultrasound could empower those providers to manage difficult removals without delay.

### Comparison with other localization methods

It is worth noting that alternative imaging modalities, such as palpation-guided X-ray or MRI, have roles mainly when ultrasound fails [3]. X-ray has the advantage of being quick and widely available, but it lacks real-time guidance and is only useful for radiopaque implants. MRI can locate even non-radiopaque implants with excellent tissue contrast, but it is costly and not practical as a first-line tool. Our case reinforces that ultrasound (whether conventional or portable) should remain the first-line and often the only modality needed in the vast majority of cases. The handheld ultrasound devices simply make this first-line tool more readily deployable. In rare scenarios of intravascular migration to the lung, ultrasound cannot reach that location—chest X-ray or CT is required. However, those are beyond the scope of routine clinic practice and fortunately extremely uncommon (roughly 1 per 100,000 insertions) [9]. The mainstay for non-palpable implants in the arm remains ultrasound, and making ultrasound more accessible via portable handheld devices is a logical advancement.

In summary, this expanded case report demonstrates that a silicon chip-based handheld ultrasound was effectively used to detect and facilitate removal of a non-palpable contraceptive implant. The handheld ultrasound’s performance was comparable to a standard ultrasound device in this context, marking an important step in point-of-care gynecologic imaging. For obstetricians and gynecologists, the ability to carry out immediate ultrasound assessments—whether for a lost implant, an IUD localization, or a quick pregnancy check—is increasingly within reach thanks to these devices. The case adds to the optimism that incorporating new ultrasound technology can enhance patient care in family planning. Future research and accumulated experience will further clarify the role of handheld ultrasound devices in improving outcomes for women with non-palpable contraceptive implants.

## Conclusion

Non-palpable contraceptive implants pose a significant clinical challenge, but advances in ultrasound technology are providing new solutions. This case report documents the first use of a semiconductor-based, portable handheld ultrasound device to successfully locate an impalpable etonogestrel implant, enabling its safe removal. The handheld ultrasound produced imaging of the implant on par with a standard device, lacking only some acoustic shadowing but otherwise clearly delineating the device. The case underscores several key points for clinicians:

- Ultrasound remains the gold standard for localizing contraceptive implants that cannot be found by touch, and it should be attempted before more invasive or expensive methods.
- Handheld, chip-based ultrasound devices are a viable alternative to standard ultrasound devices for this application, with the benefit of being usable in a wider range of clinical settings.
- Incorporating handheld ultrasound devices into clinical practice can streamline the management of non-palpable implants, reducing the need for referrals and multiple visits, and potentially improving patient satisfaction and outcomes.
- Training and experience in point-of-care ultrasound for gynecologic applications will be important to maximize the benefits of this technology.
- Further studies should be conducted to validate the accuracy of handheld ultrasound devices in implant localization across larger populations and to develop guidelines on its use.

For obstetricians and gynecologists, staying abreast of such technological developments is crucial. As shown in this report, the marriage of cutting-edge ultrasound-on-a-chip technology with a common clinical scenario (difficult implant removal) exemplifies how innovation can directly solve clinical problems. With appropriate training and adoption, handheld ultrasound devices may soon become an invaluable tool in the repertoire of family planning and reproductive health services, improving care for women worldwide.

## Data Availability

All data generated or analyzed during this study are included in this published article.
